# *Notes from the Field*: Firearm Suicide Rates, by Race and Ethnicity — United States, 2019–2022

**DOI:** 10.15585/mmwr.mm7248a3

**Published:** 2023-12-01

**Authors:** Wojciech Kaczkowski, Scott R. Kegler, May S. Chen, Marissa L. Zwald, Deborah M. Stone, Steven A. Sumner

**Affiliations:** ^1^Division of Injury Prevention, National Center for Injury Prevention and Control, CDC; ^2^Division of Violence Prevention, National Center for Injury Prevention and Control, CDC.

Suicide, including firearm suicide, remains a substantial public health concern in the United States. During the previous 2 decades, overall suicide rates and firearm suicide rates have risen by approximately one third, approaching 50,000 overall suicides during 2022, including approximately 27,000 firearm suicides (*1*). Firearm suicides account for approximately one half of all suicides, and this proportion has been increasing (*2*,*3*). This analysis includes national firearm suicide data from 2019 through the end of 2022, categorized by race and ethnicity, presented both annually and by month (or quarterly) to track subannual changes. 

## Investigation and Outcomes

National Vital Statistics System mortality data for 2019–2021 (final) and 2022 (provisional), stratified by race and ethnicity, were obtained from CDC WONDER* (*1*). Corresponding population estimates were obtained from the U.S. Census Bureau.^†^ Annual and monthly crude rates were calculated by race and ethnicity, with all rates expressed per 100,000 person-years. Because subannual data for non-Hispanic American Indian or Alaska Native (AI/AN) persons involve small monthly counts, these data are presented by quarter. This activity was reviewed by CDC, deemed not research, and was conducted consistent with applicable federal law and CDC policy.^§^

The annual U.S. firearm suicide rate increased approximately 11% from 7.3 per 100,000 during 2019 to 8.1 during 2022 (Table), the highest documented level since at least 1968 (the earliest year for which data are available in CDC WONDER). Firearm suicide rates increased in all racial and ethnic groups from 2019 through 2022, but the magnitude of increase differed among groups (Supplementary Figure; https://stacks.cdc.gov/view/cdc/135612). For example, whereas non-Hispanic White persons experienced the highest overall rate (11.1 during 2022), this rate represented a 9% increase from 10.2 during 2019. The largest rate increase (66%) occurred among AI/AN persons, among whom the firearm suicide rate increased from 6.4 during 2019 to 10.6 during 2022. During 2022, rates were lower among non-Hispanic Black or African American and Hispanic or Latino persons (5.3 and 3.3, respectively); rates in these groups increased 42% and 28%, respectively, from 2019 through 2022. The lowest firearm suicide rates were among non-Hispanic Asian or Pacific Islander^¶^ persons, increasing 10%, from 1.7 during 2019 to 1.9 during 2022.

**TABLE:** Annual firearm suicide rates and counts, by race and ethnicity — United States, 2019–2022

Race and ethnicity*	Rate^†^ (no.)			
	2019	2020	2021	2022
AI/AN, NH	6.4 (154)	9.3 (225)	10.0 (241)	10.6 (256)
A/PI, NH	1.7 (337)	1.7 (343)	1.9 (379)	1.9 (395)
Black or African American, NH	3.8 (1,546)	4.3 (1,784)	5.2 (2,165)	5.3 (2,237)
White, NH	10.2 (20,090)	10.0 (19,851)	10.8 (21,197)	11.1 (21,726)
Hispanic or Latino	2.5 (1,534)	2.9 (1,790)	3.3 (2,037)	3.3 (2,073)
**Overall^§^**	**7.3 (23,941)**	**7.3 (24,292)**	**7.9 (26,328)**	**8.1 (27,024)**

**Sources:** CDC WONDER; U.S. Census Bureau (NC-EST2020-ALLDATA; NC-EST2022-ALLDATA).

**Abbreviations:** AI/AN = American Indian or Alaska Native; A/PI = Asian or Pacific Islander; NH = non-Hispanic.

* Persons within some racial and ethnic groups, particularly AI/AN persons, might be undercounted because of misclassification. https://www.cdc.gov/nchs/data/series/sr_02/sr02_172.pdf; https://www.cdc.gov/nchs/data/nvsr/nvsr70/NVSR70-12.pdf

^† ^Crude rates represent the number of firearm suicides per 100,000 person-years.

^§^ Rates and numbers for the “Overall” category include the non-Hispanic multiple-race population grouping, not shown separately in the table.

## Preliminary Conclusions and Analysis

Firearm suicide rates increased in all racial and ethnic groups from 2019 through 2022. Multiple social and structural factors likely contributed to these increases. The large increase in the AI/AN rate might reflect systematic inequities, such as in mental health care access or unemployment, worsened by the COVID-19 pandemic (*4*); the pandemic might also have exacerbated known risk factors related to social isolation, relationship stressors, and substance use, broadly affecting observed trends (*5*).

The persistent upward trend in firearm suicide rates since 2020 across all racial and ethnic groups, coupled with the unprecedented high rates during 2022, highlight the need for continued prevention efforts. Public health organizations can facilitate collaborative cross-sector efforts to implement comprehensive, evidence-based prevention strategies, which are detailed in CDC’s Suicide Prevention Resource for Action.** Potential approaches to reducing firearm suicides include promoting secure firearm storage and counseling mental health and social services providers on access to lethal means. Other strategies to reduce suicide risk include fostering positive social connections, identifying and supporting persons at risk, and addressing underlying inequities in economic security and housing. For persons who are struggling or in crisis, help is available through the 988 Suicide and Crisis Lifeline (available at https://www.988lifeline.org/ or by texting or calling 988).

## Footnotes

* Persons within some racial and ethnic groups, particularly AI/AN persons, might be undercounted because of misclassification. https://www.cdc.gov/nchs/data/series/sr_02/sr02_172.pdf; https://www.cdc.gov/nchs/data/nvsr/nvsr70/NVSR70-12.pdf

† Monthly population estimates, by age, sex, race, and Hispanic origin, April 1, 2010–July 1, 2020; NC-EST2020-ALLDATA were used for January 2019–March 2020 estimates (https://www.census.gov/programs-surveys/popest/technical-documentation/research/evaluation-estimates/2020-evaluation-estimates/2010s-national-detail.html). Monthly population estimates by age, sex, race, and Hispanic origin, April 1, 2020–July 1, 2022, with short-term projections to December 2023; NC-EST2022-ALLDATA were used for April 2020–December 2022 estimates (https://www.census.gov/data/tables/time-series/demo/popest/2020s-national-detail.html) (Accessed June 29, 2023).

§ 45 C.F.R. part 46, 21 C.F.R. part 56; 42 U.S.C. Sect. 241(d); 5 U.S.C. Sect. 552a; 44 U.S.C. Sect. 3501 et seq.

¶ Data for non-Hispanic Asian and non-Hispanic Native Hawaiian or other Pacific Islander persons were combined to form a single non-Hispanic Asian or Pacific Islander reporting category.

**
https://www.cdc.gov/suicide/resources/prevention.html

## References

[1] CDC. National Center for Health Statistics mortality data on CDC WONDER. Atlanta, GA: US Department of Health and Human Services, CDC. Accessed August 11, 2023. https://wonder.cdc.gov/mcd.html

[2] Kegler SR, Simon TR, Zwald ML, Vital signs: changes in firearm homicide and suicide rates—United States, 2019–2020. MMWR Morb Mortal Wkly Rep 2022;71:656–63. 10.15585/mmwr.mm7119e1 35550497 PMC9098246

[3] Simon TR, Kegler SR, Zwald ML, Notes from the field: increases in firearm homicide and suicide rates—United States, 2020–2021. MMWR Morb Mortal Wkly Rep 2022;71:1286–7. 10.15585/mmwr.mm7140a4 36201375 PMC9541032

[4] Stone D, Trinh E, Zhou H, Suicides among American Indian or Alaska Native persons—National Violent Death Reporting System, United States, 2015–2020. MMWR Morb Mortal Wkly Rep 2022;71:1161–8. 10.15585/mmwr.mm7137a1 36107803 PMC9484806

[5] Czeisler MÉ, Lane RI, Petrosky E, Mental health, substance use, and suicidal ideation during the COVID-19 pandemic—United States, June 24–30, 2020. MMWR Morb Mortal Wkly Rep 2020;69:1049–57. 10.15585/mmwr.mm6932a1 32790653 PMC7440121

